# Uncoupling of Elastin Complex Receptor during *In Vitro* Aging Is Related to Modifications in Its Intrinsic Sialidase Activity and the Subsequent Lactosylceramide Production

**DOI:** 10.1371/journal.pone.0129994

**Published:** 2015-06-18

**Authors:** Amandine Scandolera, Fanja Rabenoelina, Carine Chaintreuil, Anthony Rusciani, Pascal Maurice, Sébastien Blaise, Béatrice Romier-Crouzet, Hassan El Btaouri, Laurent Martiny, Laurent Debelle, Laurent Duca

**Affiliations:** Laboratoire Signalisation et Récepteurs Matriciels (SiRMa), UMR CNRS/URCA 7369, SFR CAP Santé, Université de Reims Champagne Ardenne, Faculté des Sciences, Reims, France; Central Michigan University School of Medicine, UNITED STATES

## Abstract

Degradation of elastin leads to the production of elastin-derived peptides (EDP), which exhibit several biological effects, such as cell proliferation or protease secretion. Binding of EDP on the elastin receptor complex (ERC) triggers lactosylceramide (LacCer) production and ERK1/2 activation following ERC Neu-1 subunit activation. The ability for ERC to transduce signals is lost during aging, but the mechanism involved is still unknown. In this study, we characterized an *in vitro* model of aging by subculturing human dermal fibroblasts. This model was used to understand the loss of EDP biological activities during aging. Our results show that ERC uncoupling does not rely on Neu-1 or PPCA mRNA or protein level changes. Furthermore, we observe that the membrane targeting of these subunits is not affected with aging. However, we evidence that Neu-1 activity and LacCer production are altered. Basal Neu-1 catalytic activity is strongly increased in aged cells. Consequently, EDP fail to promote Neu-1 catalytic activity and LacCer production in these cells. In conclusion, we propose, for the first time, an explanation for ERC uncoupling based on the age-related alterations of Neu-1 activity and LacCer production that may explain the loss of EDP-mediated effects occurring during aging.

## Introduction

Elastin is an extracellular matrix protein important for the structural integrity and function of tissues undergoing reversible extensibility or deformability, such as lungs, arteries and skin [1,2]. This protein, deposited during the early stages of life [3], is extremely stable and resistant. With a typical half-life estimated at 70 years, elastin is present for life and undergoes virtually no turnover [4]. Nevertheless, during aging, mechanical stress and elastase activities contribute to the fragmentation of this macropolymer into elastin-derived peptides (EDP) [5].

While elastin is devoid of biological activity, EDP have been shown to regulate several biological processes such as immune response [6,7], chemoattraction [8], cell proliferation [9], proteases expression [10,11], calcium modulation [12], NO (Nitric Oxide) production [13,14], matrix synthesis [10,14–16] and cell migration [15,17]. As a consequence, EDP, which concentration increases in blood circulation with age [6], are thought to be involved in several aging-related pathologies such as atherosclerosis [18] and cancer [15,16,19–21].

Biologically active EDP possess a GXXPG consensus sequence adopting a type VIII β-turn structure [22,23] involved in their binding to the elastin receptor complex (ERC) [24]. This receptor contains three subunits: a peripheral 67-kDa protein named elastin-binding protein (EBP, accession number P16278-2), and two membrane-associated proteins, the protective protein/cathepsin A (PPCA, 55-kDa, accession number P10619) and neuraminidase-1 (Neu-1, 61-kDa, accession number Q99519) [25]. EBP is an enzymatically inactive spliced variant of lysosomal β-galactosidase [26,27]. It possesses two functional binding sites: the elastin site on which EDP bind triggering signaling pathways, and a galactolectin site whom occupancy by galactosugars induces EDP release, dissociation of the complex and signal loss [25].

EDP binding to EBP leads to Neu-1 activation and lactosylceramide (LacCer) generation through the desialylation of its substrate, the GM_3_ ganglioside [28]. This lipid conversion further induces the activation of the extracellular signal-regulated kinase 1/2 (ERK1/2) pathway [29] *via* a mechanism requiring both p110γ /Raf-1 and PKA/B-Raf signaling [30,31].

The biological effects induced by EDP are modified with aging. Varga *et al* and Faury *et al* suggested that the function and availability of the ERC could change with aging in leukocytes [32,33] and in arteries [13]. These effects were not explained by modifications of EBP expression and/or affinity [34] but, possibly, by altered receptor functioning and/or alteration of actors of the downstream signaling pathways [35]. Until now, the cause of this uncoupling is still a matter of speculation.

As ERC is involved in EDP-induced signal transduction [7,29,36,37] but also in elastogenesis through tropoelastin binding and secretion [25,38,39], it is of high interest to understand the mechanism leading to its age-related uncoupling.

In this study, we demonstrate that ERC uncoupling observed in an *in vitro* model of fibroblast aging is not linked to modifications of its mRNA, protein levels, or membrane targeting. However, we evidence that EDP-triggered lactosylceramide production and Neu-1 sialidase activity is reduced in aged cells. We also showed that these processes are associated with a drastic elevation of the basal catalytic activity of Neu-1 to a level being no more increasable by EDP stimulation. We therefore propose that ERC uncoupling could originate from its unability to regulate Neu-1 activity and lactosylceramide production.

## Materials and Methods

### Reagents

Elastin peptides were prepared as described by Rusciani *et al* [28]. Briefly, insoluble elastin was prepared from bovine ligamentum nuchae by hot alkali treatment. Comparing its amino acid composition to that predicted from the elastin gene product assessed its purity. Soluble elastin peptides were further obtained from insoluble elastin by organo-alkaline hydrolysis. This was achieved using 1 M KOH in 80% aqueous ethanol. The obtained mixture of elastin peptides is termed κ-elastin (kE) and exhibits the same biological properties as physiological elastin hydrolysates obtained using elastase [10]. Indeed, this mixture of EDP has been shown to contain several peptides harboring the bioactive GxxPG motifs [29].

The synthetic peptides, VGVAPG and its VVGPGA scramble, were obtained from Genecust (purity >98%) (Dudelange, Luxembourg). Dulbecco’s Modified Eagle’s Medium (DMEM), antibiotics and fungizone were purchased from Life Technologies (Saint Aubin, France). Fetal calf serum (FCS) was obtained from PAA Laboratories (Velizy-Villacoublay, France). BCA assay Protein Quantification Kit and Uptiblue reagent were from Interchim (Montluçon, France). All generic chemical products were obtained from Sigma-Aldrich (Saint-Quentin Fallavier, France). 2,3-dehydro-2-deoxy-N-acetylneuraminic acid (DANA) was used as a Neu-1 inhibitor [29,40] and was purchased from Merck (Molsheim, Fance). The Image-iT LIVE Green Reactive Oxygen Species Detection Kit (Molecular Probes) was obtained from Invitrogen (Cergy-Pontoise, France).

### Antibodies

Alexafluor-568 goat anti-rabbit antibodies were from Molecular Probes (Saint Aubin, France). Anti-phospho-ERK1/2, anti-actin and anti-Neu-1 (H-300) polyclonal antibodies were obtained from Santa Cruz (distributed by Tebu, Le Perray en Yvelines, France). Anti-ERK1/2 antibody was purchased from Cell Signaling Technology Inc. (Beverly, MA, distributed by Ozyme, Saint Quentin en Yvelines, France). FITC-conjugated anti-CD17 antibody (anti-LacCer) and FITC-labeled isotype control were from Ancell, distributed by Covalab (Villeurbanne, France). Fluorochrome-coupled anti-rabbit or anti-mouse secondary antibodies were obtained from Invitrogen. HRP-coupled anti-mouse antibody was purchased from Life Technology. HRP-coupled anti-goat antibody was obtained from Sigma-Aldrich.

### Ethics statement

Human skin fibroblasts were established from explants of human adult skin biopsies obtained from informed healthy volunteers (aged 33–44 years) who have given their written consent. The experiments were conducted according with the recommendations of "Le Centre National de la Recherche Scientifique" (CNRS, France) that has specifically approved this study. Collection and utilization of human skin biopsies were approved by the Institutional Review Board of the Reims University Hospital (CHU de Reims).

### Fibroblasts isolation, cell culture and treatments

Human dermal fibroblasts were isolated from skin biopsies of healthy subjects and were obtained using cell culture medium selection [41]. Briefly, the hypodermis was mechanically eliminated and explants were cut into 1 mm^2^ pieces. Skin fragments were then cultivated at 37°C/5% CO_2_ in 75 cm^2^ cell culture dishes containing 10 mL of DMEM medium supplemented with 20% FCS, 1% antibiotics (penicillin, streptomycin) and 1% fungizone. The medium was changed every two days. After 4 weeks of culture, fibroblasts have covered all the surface of the culture dishes and were trypsined. Cells were cultured at 37°C/5% CO_2_ in monolayers in DMEM containing 1g/L of glucose, Glutamax I and pyruvate supplemented with 10% FCS. Subcultures 3 to 10 were used in this study.

Before all experiments, cells were starved for 18 hours by FCS privation in order to synchronize them. Fibroblasts were then stimulated or not with 50 μg/mL EDP or 200 μg/mL VGVAPG or VVGPGA peptides in basal medium. To inhibit Neu-1 activity, fibroblasts were pre-incubated for 1 h with 400 μM DANA then stimulated as described above. Stimulation was stopped by adding cold phosphate buffered saline (PBS) containing 50 μM Na_3_VO_4_.

### Acid β-galactosidase assay

The detection of β-galactosidase activity at pH6 is a specific biomarker of cellular senescence [42,43]. Cells (3.10^5^) were seeded in a 6-well plate in medium containing 10% FCS and 1% antibiotics. After 48 hours of culture followed by an 18h-serum starvation period β-galactosidase staining was performed using a β-galactosidase staining kit (Cell Signaling, Danvers USA). Cells were washed twice in PBS then fixed for 10 minutes at room temperature with 2% formaldehyde and 0.2% glutaraldehyde in PBS. After two washes in PBS, cells were incubated overnight at 37°C with β-galactosidase staining solution containing 1X staining solution (40 mM citric acid/sodium phosphate pH6, 150 mM NaCl and 2 mM MgCl_2_), 1X staining solution supplement A (5 nM potassium ferrocyanide), 1X staining solution supplement B (5 nM potassium ferricyanide) and 1 mg/mL X-gal (5-bromo-4-chloro-3-indolyl-β-D-galactopyranoside). Three pictures were taken per well and the stained cells were counted.

### Proliferation assays

Fibroblasts (1.10^5^) were seeded in 24-well plates in complete DMEM medium. After 48 hours of culture, cells were starved during 18 hours by serum privation then stimulated for 48h with 50 μg/mL of EDP or 200 μg/mL VGVAPG or scramble peptide in basal medium. Cellular proliferation was analyzed by two different approaches: manual counting after Trypan blue staining and by colorimetry with the Uptiblue reagent. For manual counting, cells were detached by trypsin incubation, stained using Trypan Blue then counted using a KOVA cell. As described by the manufacturer, the UptiBlue method uses a colorimetric growth indicator based on the detection of metabolic activity. It depends on a redox indicator that changes color in response to chemical reduction of growth medium resulting from cell growth, allowing to determine cell proliferation [44]. After the stimulation period, fibroblasts were incubated with the Uptiblue reagent for 4 hours at 37°C. Cell proliferation was then evaluated by absorbance at 560 nm.

### Reactive oxygen species (ROS) detection

Fibroblasts (1.10^5^) were seeded in a 24-well plate in complete DMEM medium. After 48 hours of culture followed by an 18h serum starvation period, cells were stimulated by 50 μg/mL EDP or 200 μg/mL VGVAPG or VVGPGA for 30 minutes. ROS production was determined using the Image-iT LIVE Green Reactive Oxygen Species Detection Kit according to the manufacturer’s instructions. ROS were detected after incubation for 30 minutes in darkness with 25 μM carboxy-2',7'-dichlorodihydrofluorescein diacetate, a chemically reduced analogue of fluorescein used as an indicator of ROS production in cells. Cells were then washed twice in PBS and oxidation products were detected by fluorescence (excitation at 495 nm, emission at 529 nm).

### MMP-1 ELISA Assay

Fibroblasts (3.10^5^) were seeded in a 6-well plate in DMEM medium containing 10% FCS and 1% antibiotics. After 48h of culture, cells were serum-starved for 18h then stimulated by 50 μg/mL EDP or 200 μg/mL VGVAPG or VVGPGA peptide during 24 hours. The supernatants were collected and MMP-1 secretion was detected by ELISA (Calbiochem, Darmstadt, Germany). Briefly, samples (standards and conditioned mediums) were incubated for 2 hours at room temperature in a 96-well plate pre-coated with MMP-1 antibodies. After washes with PBS, HRP-conjugated MMP-1 was added during 1 hour at room temperature. Bound MMP-1 was detected by adding the chromogenic substrate tetra-methylbenzidine for 30 minutes at room temperature in darkness. The reaction was stopped by addition of 2.5 N sulfuric acid. MMP-1 detection was analyzed by reading absorbance at 450 nm using a micro-plate reader (TECAN) coupled to the Magellan software.

### Western blotting

Fibroblasts (3.10^5^) were seeded in a 6-well plate in complete DMEM medium. After 48h of culture, cells were serum-starved for 18h then stimulated with 50 μg/mL EDP for 24 hours. Reaction was stopped by adding ice-cold PBS containing 50 μM Na_3_VO_4_. Fibroblasts were then washed twice in ice-cold PBS containing 50 μM Na_3_VO_4_, scrapped and sonicated in lysis buffer (PBS, pH7.4, 0.5% Triton X-100, 80 mM glycerophosphate, 50 mM EGTA, 15 mM MgCl_2_, 1 mM Na_3_VO_4_ and protease inhibitor cocktail (Sigma-Aldrich)). Insoluble material was removed by centrifugation (20000*g*, 45 min, 4°C). Protein concentration was determined by the BCA assay Protein Quantification Kit. Equal amounts of proteins were heated for 5 minutes at 100°C in sample buffer and separated by SDS-PAGE under reducing conditions and transferred to nitrocellulose membranes. Membranes were saturated in blocking buffer containing 5% milk (w/v) in TBS-T buffer (50 mM Tris, pH7.5, 150 mM NaCl, 0.1% (v/v) Tween 20) for 1 hour at room temperature and incubated overnight at 4°C with anti-phospho-T202/Y204-ERK1/2, anti-ERK1/2, anti-Neu-1, anti-PPCA (kindly provided by Prof. A. d’Azzo, Memphis, USA) or anti-β-actin (1/1000) antibodies in blocking buffer. After five washes with TBS-T buffer, membranes were incubated for 1 hour at room temperature with HRP-coupled anti-rabbit, -mouse or -goat secondary antibodies (1/5000) (Cell Signaling) in blocking buffer. Immuno-complexes were detected by chemiluminescence and quantified by Quantity One software (Biorad).

### Real Time-qPCR

Fibroblasts (3.10^5^) were seeded in a 6-well plate in complete DMEM medium. After 48h of culture, cells were serum-starved for 18h then stimulated at 50 μg/mL EDP for 24 hours. Total mRNA were extracted by Trizol then separated from others cellular materials by chloroform/isoamyl alcohol (24:1) and centrifugated (12000*g*, 4°C, 15 min). Total mRNA were precipitated with isopropanol then washed with 75% ethanol and resuspended in water. The quantity of RNA was determined by spectroscopy and their quality was evaluated by migration on agarose gel. 250 ng of total mRNA were reverse-transcribed using VERSO cDNA kit (Thermoscientific, USA) according to the manufacturer’s instructions. RT-qPCR primers were designed according to human sequences encoding for EBP, β-Gal, NEU-1, PPCA, RS18 and RPL32 (Table 1).

**Table 1:** 

Primers		Sequence	Amplicon (pb)
**RS18**	forward	5’-GCAGAATCCACGCCAGTACAA-3’	208
	reverse	5’-GCCAGTGGTCTTGGTGTGCT-3’	
**RPL32**	forward	5’-CATTGGTTATGGAAGCAACAAA-3’	150
	reverse	5’-TTCTTGGAGGAAACATTGTGAG-3’	
**EBP**	forward	5’-TCCAGACATTACCTGGCAGCT-3’	111
	reverse	5’-ATGTTGCTGCCTGCACTGTT-3’	
**β-gal**	forward	5’-GCTGGTTATCCTGAGGCCC-3’	104
	reverse	5’-CGGAGGAGCGGAGAAGAATA-3’	
**NEU-1**	forward	5’-AATGCCCGAAACCAGAACAAC-3’	239
	reverse	5’-CGCCATGAGGTACCATTGCT-3’	
**PPCA**	forward	5’-AATCTCTATGCCCCGTGTGCT-3’	102
	reverse	5’-TGGCAGGCGAGTGAAGATG-3’	

All primers were synthetized by Eurogentec (Angers, France). Real-time PCR was performed using an Absolute SYBR Green Rox mix (Thermo Electron, Courtaboeuf, France), and the Chromo Four-Color-Real-Time PCR detection system (Bio-Rad, France). PCR conditions were 15 minutes at 95°C, followed by 40 cycles each consisting of 15 s at 95°C (denaturation) and 1 minute at 60°C (annealing/extension). PCR efficiency of the primer sets (EBP, β-Gal, Neu-1, PPCA, RS18, and RPL32) was controlled via the slope of a standard curve. Results were standardized to RS18 and RPL32 gene expression levels using Genex software (BioRad).

### Immunofluorescence

Fibroblasts (3.10^4^) were seeded on glass slides in 24-well plates in complete DMEM medium. After 48h of culture followed by an 18h serum starvation period, cells were stimulated or not with 50 μg/mL EDP for 30 minutes. Cells were fixed with 4% paraformaldehyde for 15 minutes and unspecific sites were saturated by 3% bovine serum albumin (BSA) (w/v) for 1 hour at room temperature. Cells were incubated overnight at 4°C with rabbit polyclonal anti-Neu-1 (1/100) or anti-PPCA (1/1000) primary antibodies diluted in 0.3% BSA, then washed twice in PBS and further incubated for 1 hour at room temperature with Alexafluor-568-coupled anti-rabbit (1/1000) secondary antibodies. After mounting with Prolong Gold Antifade reagent containing DAPI, the slides were imaged by confocal microscopy (Zeiss).

### Sialidase activity assay

Fibroblasts (2.10^6^) were seeded in Petri dishes in complete DMEM medium. After 48 hours of culture followed by a 18h serum starvation period, cells were stimulated with PBS or elastin peptides (50 μg/mL) for 30 minutes at 37°C. Cells were then washed and crude membranes were prepared in TEM buffer pH7.5 containing 75 mM Tris pH7.5, 2 mM EDTA, 12 mM MgCl_2_, 1mM Na_3_VO_4_, 10 mM NaF and protease inhibitor cocktail. Cellular extracts were sonicated then centrifuged (600*g*, 10 min, 4°C). The supernatant was collected and centrifuged again at 20000*g* for 45 minutes at 4°C. The crude membrane pellet was suspended in 200 μL of MES buffer (20 mM 2-(*N*-morpholino) ethane sulfonic acid, pH 7). 50 μg of crude membranes were deposited per well in a 24-well plate. They were stimulated in 200 μL MES buffer (pH 7) containing or not 50 μg/mL EDP and 200 μM of chromogenic substrate *para-*nitrophenyl-N-acetylneuraminic acid. After incubation for 4h at 37°C in darkness, the reaction was stopped with 300 μL of 1M Na_2_CO_3_. Finally, sialidase activity was measured by absorbance at 405 nm.

### Flow cytometry

Fibroblasts (3.10^5^) were seeded in 6-well plates in complete DMEM medium. After 48h of culture, cells were serum-starved for 18h. Fibroblasts were then preincubated with 400 μM of DANA for 1h then stimulated or not with EDP (50 μg/mL) for 30 minutes at 37°C. After stimulation, fibroblasts were washed with ice-cold PBS containing 50 μM Na_3_VO_4_ then harvested in PBS containing 10 mM EDTA at 4°C. Cells were centrifuged (400*g*, 10 min, 4°C), washed with PBS, then incubated with FITC-anti-CD17 antibody (anti-lactosylceramide) or FITC-anti-IgM control (1/25 dilution) for 20 minutes at room temperature in darkness. Cells were finally washed, centrifuged and fixed with 500 μL 1% formaldehyde before analysis with a FACS Calibur cytometer (Becton Dickinson, Le Pont de Claix, France).

### Statistical analysis

Results are expressed as mean +/- S.E.M of 3 independent experiments, each run in triplicate. Comparison between groups were made using two-tailed Student’s *t* test. The results were considered significantly different at p<0.05.

## Results

### Characterization of the *in vitro* model of fibroblast aging

Primary cell sub-cultures were used as an *in vitro* model of aging. This model has been reported to mimic cellular and gene expression modifications observed in normal *in vivo* aging while limiting inter-individual variability [45,46].

**Fig 1:**
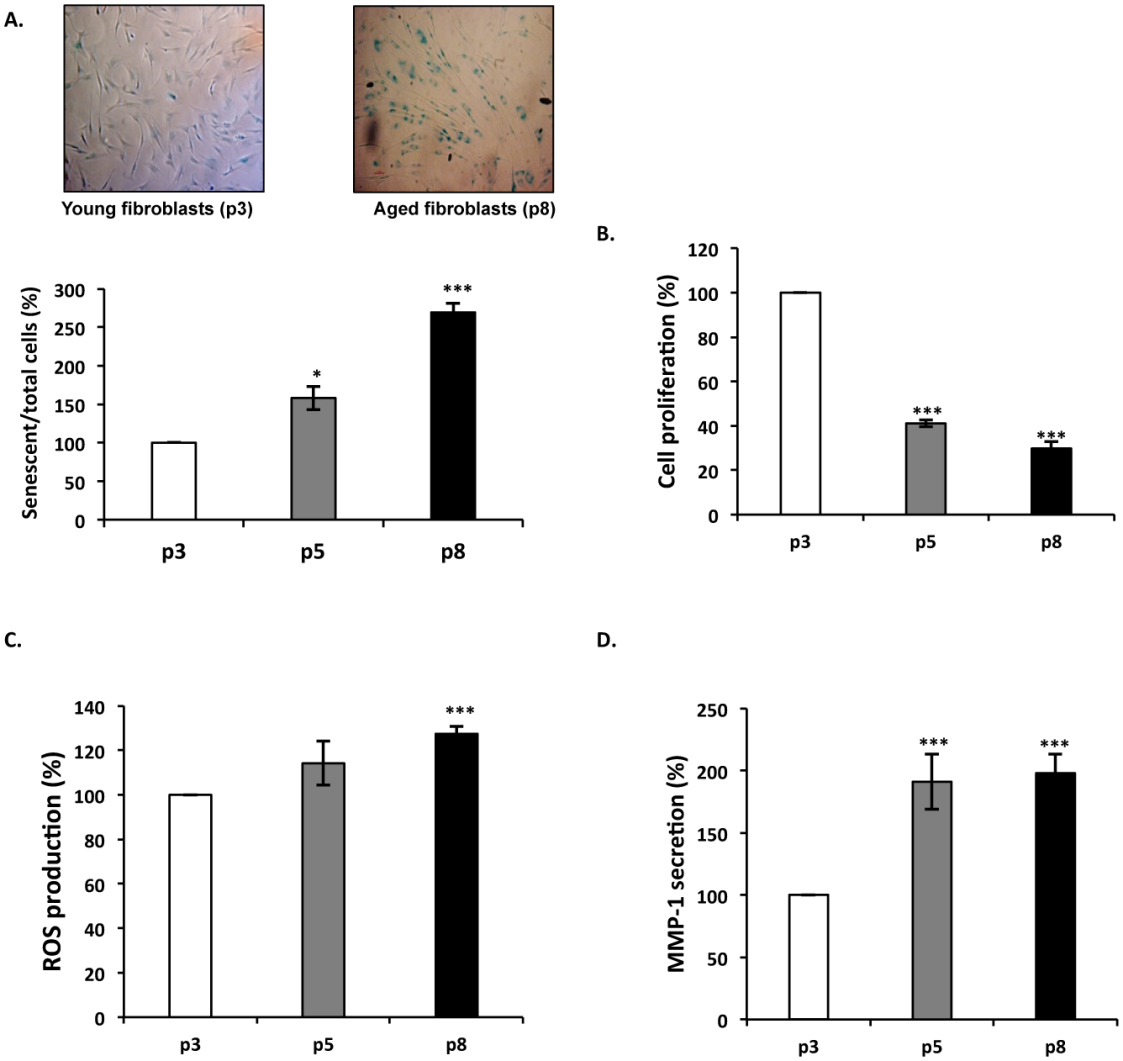
Characterization of the *in vitro* model of aging. Young, intermediate and aged fibroblasts are represented by passage 3 (white), 5 (gray) and 8 (black bars), respectively. Data shown are representative of three independent experiments. (A) Acid β-galactosidase detection during *in vitro* aging. Acid β-galactosidase positive senescent cells appear in blue. The histogram reports the senescent/total cells ratio in the three conditions. This ratio was set to 100% for p3. (B) Fibroblasts proliferation is decreased during *in vitro* aging. Cells were incubated with the Uptiblue reagent and proliferation was evaluated by measuring the absorbance at 560nm. The results are expressed as a percentage of p3 condition value. (C) Evaluation of ROS production during *in vitro* aging. ROS production was detected by fluorescence using the Image-iT LIVE Green Reactive Oxygen Species Detection Kit. (λ _excitation_ 495nm / λ _emission_ 529nm). The results are expressed as a percentage of p3 condition value. (D) Evalution of MMP-1 secretion during *in vitro* aging. MMP-1 secretion was determined by ELISA. The results are expressed as a percentage of p3 condition value. *, *p<*0.05, ***, *p*<0.01.

Morphological changes, decreased replicative capacity, acid β-galactosidase expression [47], ROS and MMP-1 production are correlated to cellular senescence [48–50]. We therefore analyzed these parameters to characterize our model of *in vitro* aging. During the *in vitro* aging process, we observed an increased proportion of senescent cells as shown by β-galactosidase detection (Fig 1A), a decrease of fibroblast proliferation (Fig 1B), and an increase of ROS production (Fig 1C) and MMP-1 secretion (Fig 1D). These data confirmed that cellular senescence occurred in our model and validated its use for the study of *in vitro* aging. As a consequence, we considered “young” fibroblasts to be represented by cells that had undergone 3 passages (white), “intermediate” fibroblasts with 5 passages (gray) and “aged” cells those with 8 passages (black).

### Impact of aging on EDP-induced biological activities

**Fig 2:**
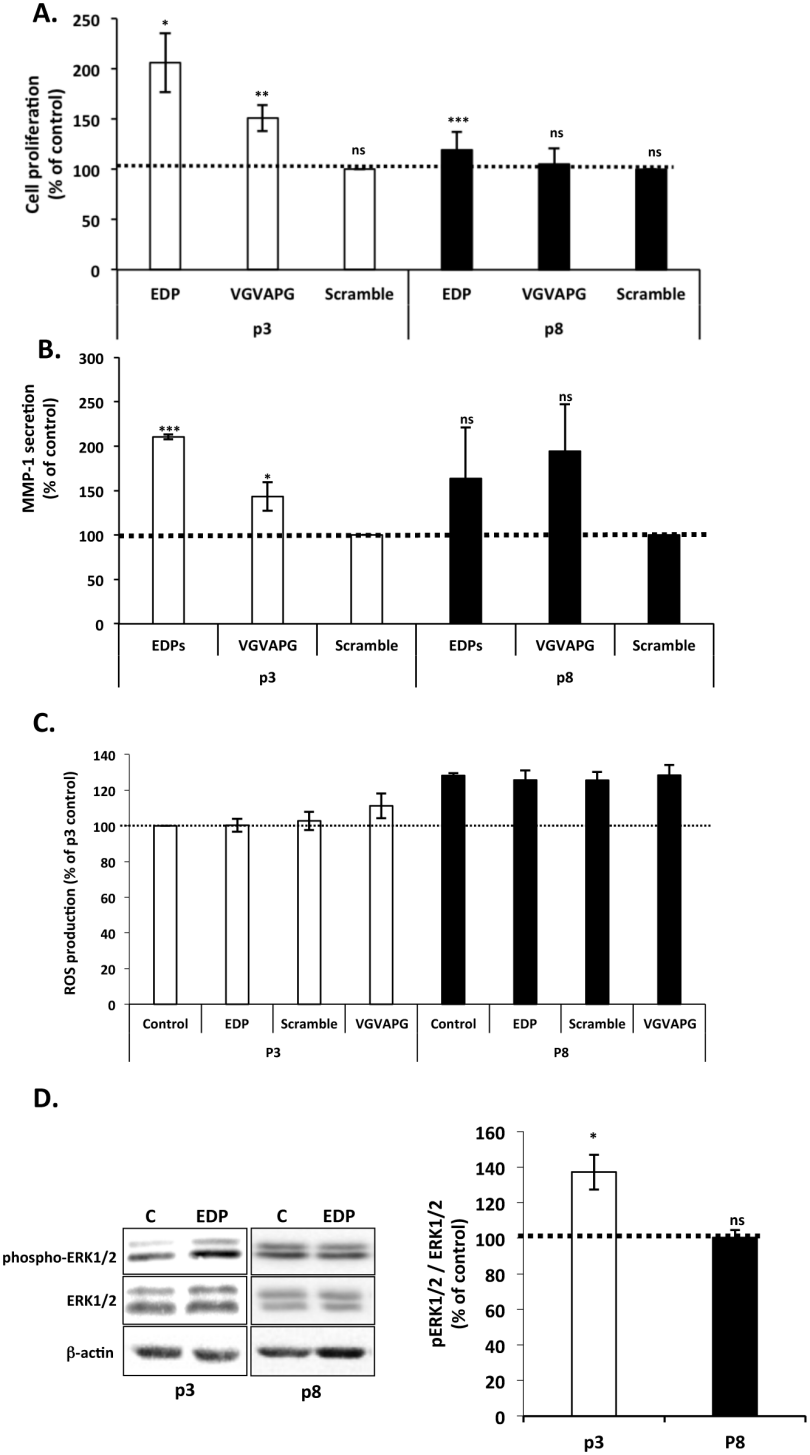
Impact of *in vitro* aging on EDP biological effects. Young and aged fibroblasts were represented by passage 3 (white) and 8 (black bars), respectively. Dotted lines (100%) represent the values of the unstimulated controls. (A) Impact of *in vitro* aging on EDP-mediated fibroblast proliferation. Cells were stimulated or not with 50 μg/mL of EDP or 200 μg/mL of scramble or VGVAPG peptides during 48h. Proliferation was analyzed by counting using Trypan Blue exclusion. (B) Impact of *in vitro* aging on EDP-induced MMP-1 secretion on fibroblasts. MMP-1 secretion was evaluated by ELISA. Cells were stimulated with 50 μg/mL of EDP or 200 μg/mL of scramble or VGVAPG peptides during 24h. (C) Impact of *in vitro* aging on EDP-induced ROS production. Cells were stimulated for 30 minutes with 50 μg/mL of EDP or 200 μg/mL of scramble or VGVAPG peptides. ROS were detected by the Image-iT LIVE Green Reactive Oxygen Species Detection Kit as described. (D) Impact of *in vitro* aging on EDP-induced ERK1/2 activation. ERK1/2 phosphorylation was analyzed by Western blotting after 30 min of stimulation with 50 μg/mL of EDP. Cells extracts were analyzed using anti-phospho-ERK1/2 (T202/Y204) and anti-ERK1/2 antibodies. The blots are presented in the left panel. The corresponding densitometric analysis is provided on the right panel. *, *p<*0.05, ** *p<*0.02, *** *p*<0.01.

EDP regulate several biological functions such as cell proliferation [9] and MMP-1 production [10,31]. These effects have been linked to ERK1/2 signaling pathway activation [29,30]. In order to analyze the impact of aging on EDP signaling, we checked the ability of EDP (kE, VGVAPG) to induce these biological activities as a function of fibroblast *in vitro* aging. As presented in Fig 2A, kE and VGVAPG increased significantly the proliferation of young (by 100%) but their effect was limited and not significant for aged cells. Scramble peptide exhibited no effect demonstrating the specificity of the stimulation. Similar results were observed on MMP-1 secretion (Fig 2B). MMP-1 secretion was increased two fold in young cells. However, the increase observed in oldest cells was not significant. We also analyzed the production of ROS following EDP treatment but we observed that it had no significant incidence (Fig 2C). The observations made with cells treated with 50 μg kE/mL were correlated with those made when cells were treated with 200 μg/mL VGVAPG (Fig 2A and 2B and 2C, p3 and p8), a well-known EBP agonist. Interestingly, a scramble of the VGVAPG sequence, VVGPGA, could not reproduce the effect of VGVAPG demonstrating the specificity of the stimulation [10,40].

As stated earlier, the biological activities of EDP involve the activation of the MAPK ERK1/2 signaling pathway [30,31]. We thus checked the ability of EDP to induce the activation of the MEK/ERK pathway in our model of *in vitro* aging. As reported in Fig 2D, we observed that EDP significantly increased by 37% the phosphorylation rate of ERK1/2 in young cells, while they had no significant effect in oldest cells (Fig 2D).

In conclusion, our results show that in our *in vitro* aging model, EDP have lost their ability to trigger biological processes in aged cells. These data suggested, as proposed by others [13,32,33], an age-related uncoupling of ERC from its signaling pathways.

### Analysis of mRNA and protein expression levels of ERC sub-units

The decrease of EDP-mediated effects does not imply a modification of EBP expression nor affinity [34]. We therefore hypothesized that a modification of the mRNA expression and protein production of the others partners of the complex (Neu-1/PPCA) could account for this decrease.

**Fig 3:**
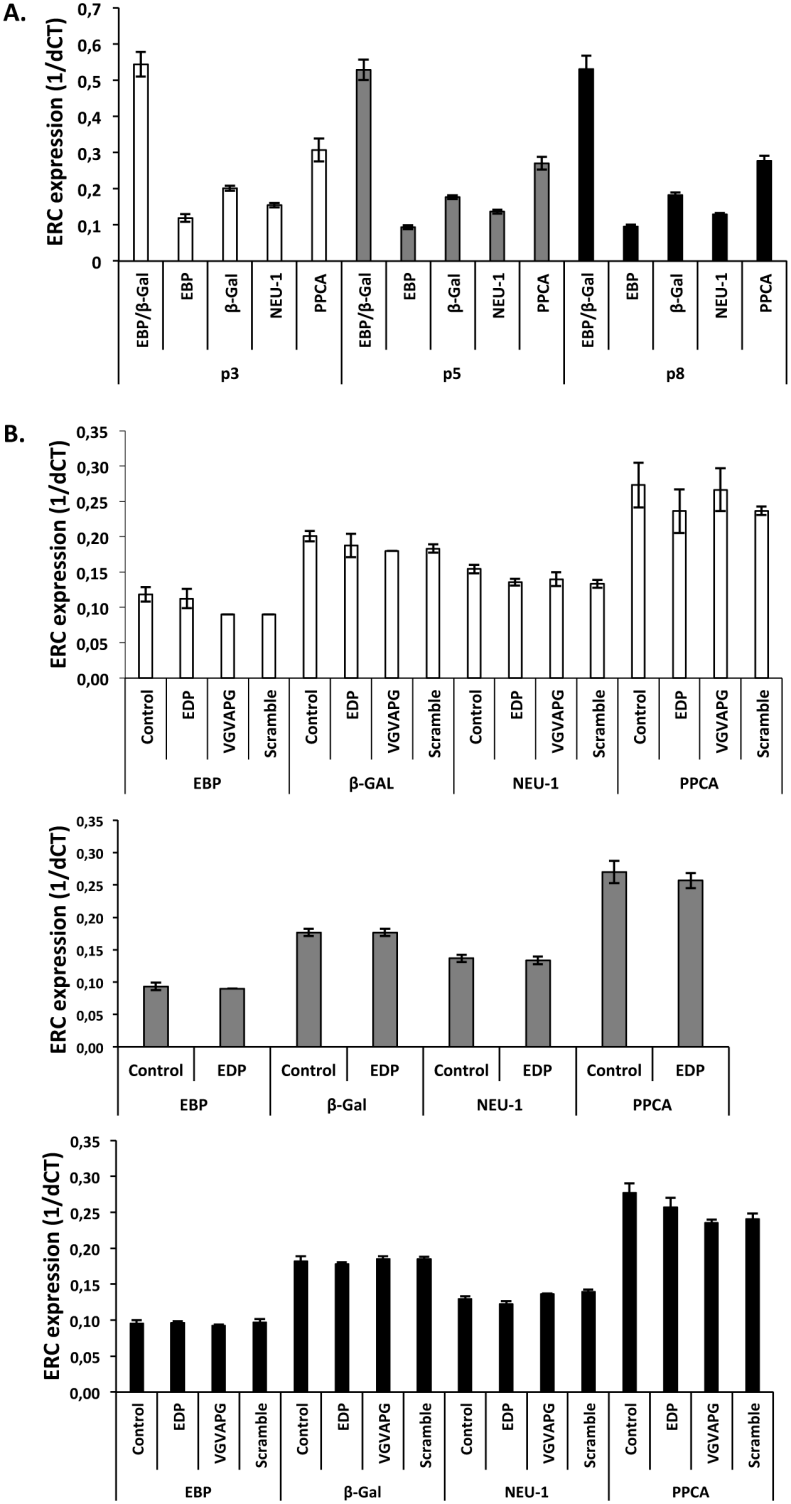
mRNA expression analysis of the three subunits of the elastin complex receptor during *in vitro* aging. Young, intermediate and aged fibroblasts were represented by passage 3 (white), 5 (gray) and 8 (black bars). (A) mRNA expression of EBP, NEU-1 and PPCA during *in vitro* aging. (B) Impact of EDP on ERC subunits expression during *in vitro* aging. mRNA levels were analyzed after 24 hours of stimulation with 50 μg/mL of EDP or 200 μg/mL of scramble or VGVAPG peptides. Results are expressed as 1/dCT. Each data are representative of three independent experiments. No significant differences were observed.

The expression level of the three ERC subunits was evaluated by RT-qPCR (Fig 3). Our data evidenced no mRNA levels changes for the EBP, PPCA and NEU-1 subunits (Fig 3A). Importantly, the EBP/β-Gal expression ratio remained unchanged throughout the *in vitro* aging process. Strikingly, the transcription levels of ERC subunits were not affected by cell aging (p3, p5 or p8) either in resting or EDP-stimulated conditions (Fig 3B). Altogether, these data indicated that age-related modifications of ERC subunits transcription could not account for the ERC uncoupling.

**Fig 4:**
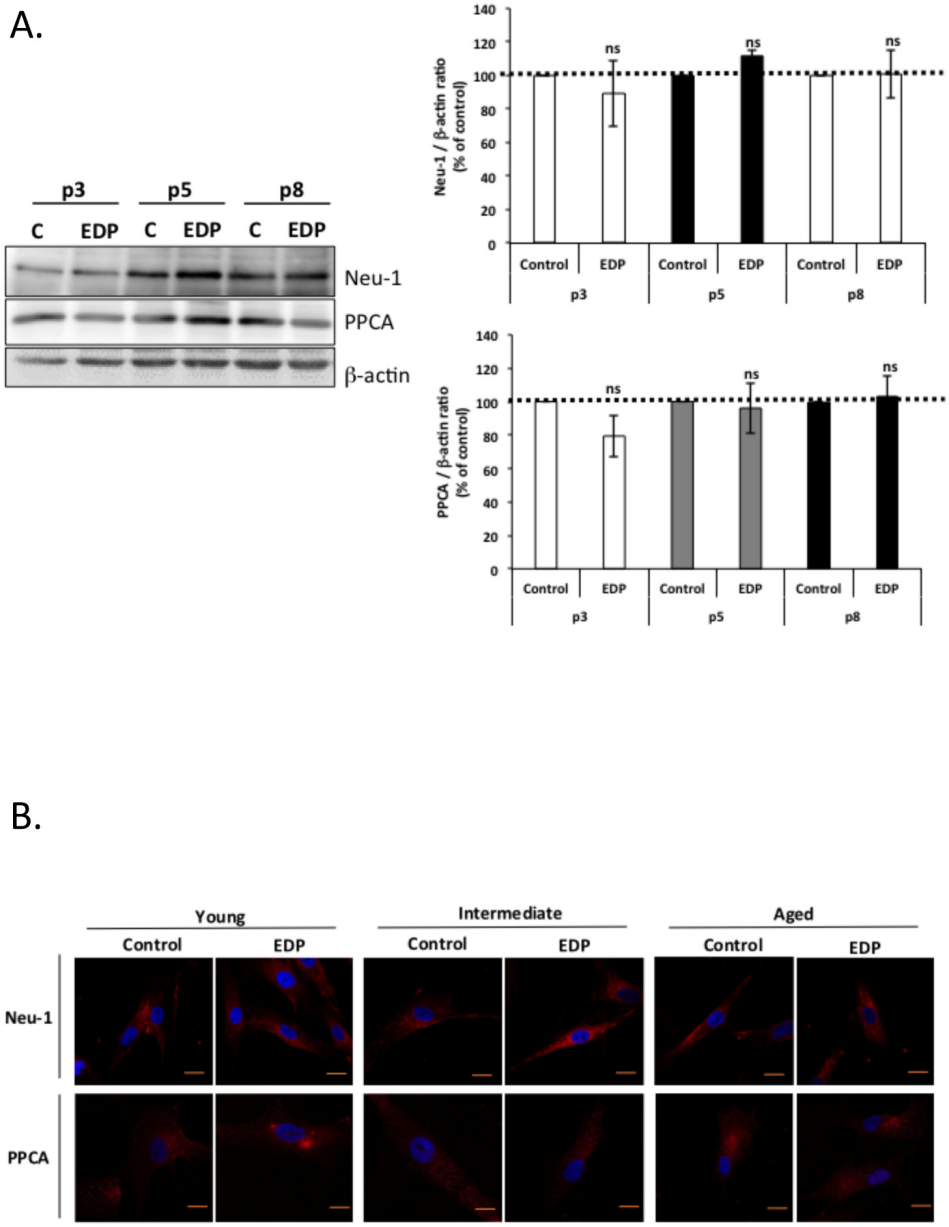
Analysis of protein expression and plasma membrane localization of Neu-1 and PPCA. Young, intermediate and aged fibroblasts were represented by passage 3 (white), 5 (grey) and 8 (black bars). (A) Analysis of Neu-1 and PPCA protein levels during aging. Cells were stimulated or not for 24 hours with 50 μg/mL of EDP before protein extraction. Neu-1 and PPCA were then analyzed by Western blotting using anti-Neu-1 and anti-PPCA antibodies. Densitometric analysis was performed using the Quantity One software. The results were expressed as the ratio Neu-1/β-actin and PPCA/β-actin and normalized to the control expressed in percent. Dotted lines (100%) represent the values of the unstimulated controls. (B) Analysis of Neu-1 and PPCA localization during aging. Neu-1 and PPCA cell localizations were analyzed by confocal microscopy. Bar, 10 μm.

As EBP protein levels showed no variation during aging [34], we focused on Neu-1 and PPCA protein levels and their localization (Fig 4). As reported in Fig 4A, neither NEU-1 nor PPCA protein levels were changed between young, intermediate and old cells. This was observed both in resting conditions and after EDP treatment. We therefore hypothesized that ERC location at the plasma membrane could be changed with age. However, immunofluorescence plasma membrane localization studies showed no changes in Neu-1 and PPCA localization during aging (Fig 4B). We therefore concluded that the protein levels and the localization of the two ERC subunits remained unchanged during aging and could not explain the age-related uncoupling of ERC. We further hypothesized that a modification of Neu-1 enzymatic activity could explain this uncoupling.

### Age-related alterations of EDP-induced sialidase activity

Gangliosides are known to modulate several signal transduction processes [51,52]. GM_3_ ganglioside is a substrate of Neu-1 and its desialylation following EDP treatment leads to LacCer production, a second messenger able to activate ERK1/2 pathway [28,29].

**Fig 5:**
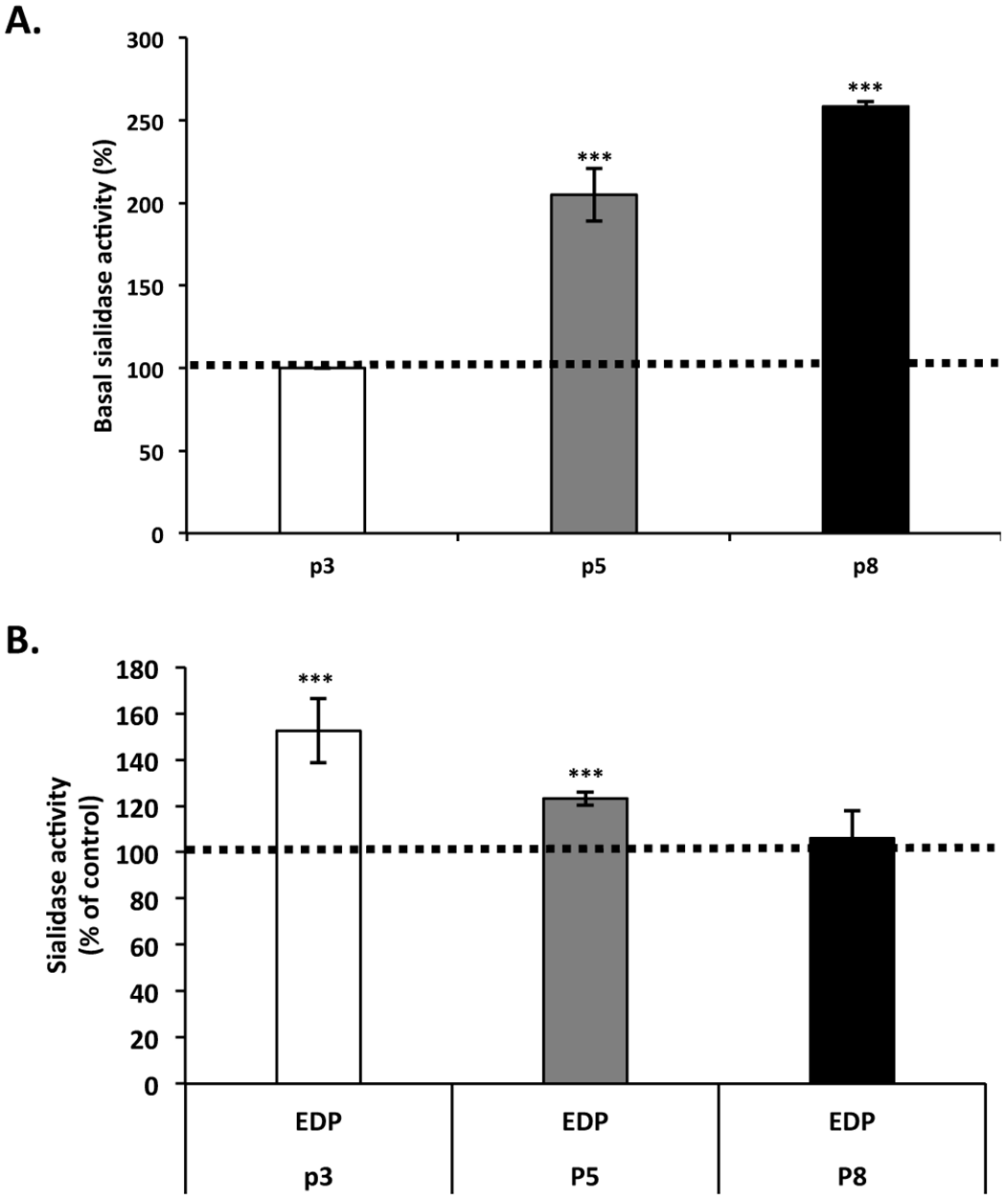
Impact of aging on EDP-induced sialidase activity. Young, intermediate and aged fibroblasts were represented by passage 3 (white), 5 (gray) and 8 (black bars). (A) Basal sialidase activity increases with age. Sialidase activity assay was performed on crude membrane using the chromogenic substrate *para-*nitrophenyl-NANA for 4 hours at 37°C. (B) EDP-mediated sialidase activity is altered with age. Cells were stimulated or not with 50 μg/mL of EDP for 30 minutes then crude membrane were prepared and used to measure sialidase activity. Histogram represent the normalized results relative to the control expressed in percent. Each data is representative of three independent experiments. ***, *p*<0.01. Dotted lines represent the values of the unstimulated controls.

In order to evaluate if Neu-1 sialidase activity could be affected by aging, we measured the effect of EDP on Neu-1-mediated sialidase activity as a function of age using a chromogenic substrate (Fig 5A). As compared to young cells, basal sialidase activity in crude membranes of intermediate and old cells showed a two-fold and three-fold increase respectively. The same assay was performed from EDP-stimulated cells. A significant sialidase activity increase (50%) was observed in young fibroblasts (Fig 5B). This increase was lower for intermediate cells (+20%) and lost in aged cells. These data suggested that uncoupling of ERC from its signaling pathway could be due to a loss of sialidase activity stimulability following EDP treatment.

### EDP-induced LacCer production as a function of age

**Fig 6:**
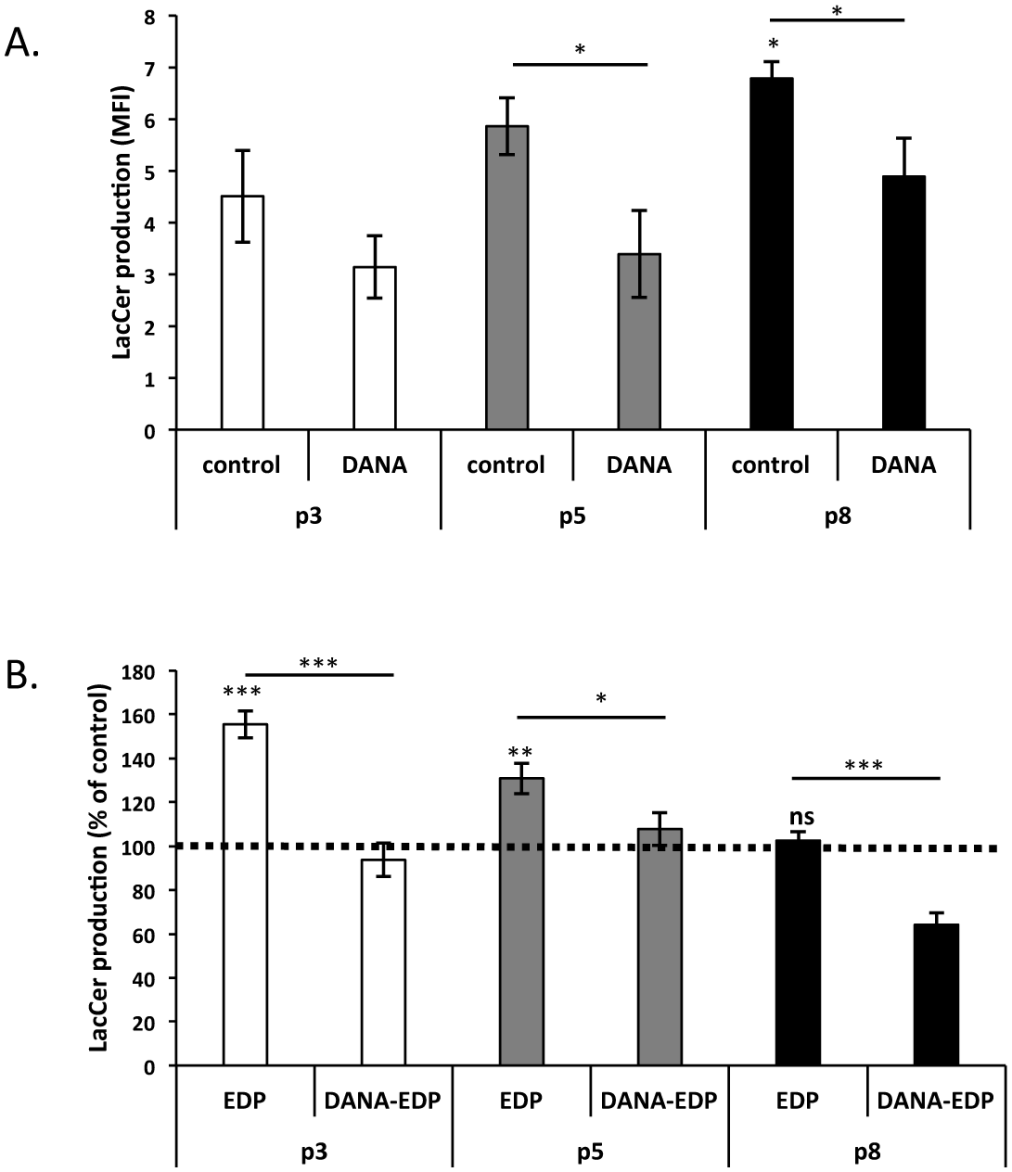
Impact of aging on EDP-induced LacCer production. Young, intermediate and aged fibroblasts were represented by passage 3 (white), 5 (gray) and 8 (black), respectively. (A) Basal LacCer production increases with age. LacCer production was detected by flow cytometry using a specific FITC-coupled anti-LacCer antibody (anti-CD17). DANA was used to study the impact of sialidase inhibition on basal production of LacCer during *in vitro* aging. (B) EDP-mediated LacCer production is altered with age. Cells were stimulated or not with 50 μg/mL of EDP for 30 minutes in the presence or not of DANA. LacCer production was then analyzed by flow cytometry as described above. Histograms represent the normalized results relative to the control expressed in percent. Each data is representative of three independent experiments. *, *p*<0.05, ***, *p*<0.01. Dotted lines represent the values of the unstimulated controls.

To evaluate if the modification observed in sialidase activity could be translated to a change in LacCer production, this lipid was measured by flow cytometry throughout the *in vitro* aging process. Our results (Fig 6A) demonstrated a progressive and significant increase of basal LacCer presence with aging. LacCer levels reached a maximal production in aged fibroblast with a 50% increase as compared to young cells. The augmentation of LacCer production correlated with the evolution of sialidase activity during *in vitro* aging and was blocked after addition of DANA, a well-known sialidase inhibitor (Fig 6B). The stimulation of cells with EDP induced a significant increase of LacCer production in young fibroblasts (+50%). This increase was comparable in intermediate cells but was lost in aged ones (Fig 6B). Moreover, we showed that DANA treatment inhibited EDP effects on LacCer production demonstrating Neu-1 involvement in LacCer production. Our results therefore suggested that ERC uncoupling is due to an age-dependent loss of EDP ability to trigger LacCer production.

## Discussion

In pathophysiological conditions, elastin is degraded and generates elastin peptides possessing several biological functions [5]. Indeed, through their capacity to regulate chemotaxis [8], proliferation [9], protease synthesis [10,14–16], and by modulating the immune system [6], EDP are involved in the progression of diverse diseases, such as cancer and cardiovascular pathologies, but also in physiological conditions like healing and aging [53]. EDP effects are triggered after their binding on the ERC, leading to the activation of Neu-1 sialidase activity. As a consequence, this enzyme converts the GM_3_ ganglioside to LacCer, a second messenger [28,29]. However, it has been described that the biological effects induced by EDP are modified with aging. Indeed, diverse group showed age-related alterations in EDP signaling [13,32,33] which did not involve a modification of EBP binding sites nor affinity [34].

We used an *in vitro* model of aging to study its impact on the biological effects of EDP. The successive subculturing is considered to be a good method to develop an *in vitro* model of aging [45,46]. This method reproduces the same age-related modifications such as decrease of replicative capacity, morphological changes [46], increase in acid β-galactosidase expression [47] and an augmentation of ROS production and MMP-1 secretion [48,49]. These senescence markers were used to characterize our model and to confirm the induction of an efficient *in vitro* aging.

EDP stimulate the proliferation of normal cells, such as fibroblasts [9,54,55] and smooth muscle cells [56], but also tumor cells from glioblastoma [57] and melanoma [15]. Consistently with literature data, our results demonstrated that EDP induce cell proliferation. However, this effect was lost in aged cells. Indeed, EDP-induced proliferation was observed in young and intermediate fibroblasts but not with aged fibroblasts. We also showed that EDP are not able to significantly trigger ROS production in young or old fibroblasts. The elastin peptides-induced ROS production has been reported in monocytes/macrophages [18]. However, inhibition of ROS production has been reported in EDP-treated neutrophils [58] suggesting that the ability of elastin peptides to trigger or not this process is cell-dependent. Our results suggest that elastin peptides are not able to regulate ROS production by fibroblasts, reinforcing this hypothesis.

MMP-1 up-regulation by EDP has been described in both normal and tumor cells, such as endothelial cells [11], fibroblasts [10], melanoma [15] and fibrosarcoma [59]. In this study, we show that this EDP-induced MMP-1 up-regulation is altered with age.

EDP-mediated MMP-1 production involves the activation of the MEK/ERK pathway through both PKA- and PI3K-dependent signaling [30]. As observed for EDP-mediated MMP-1 secretion, we showed that only young and intermediate fibroblasts could trigger ERK1/2 signaling pathway in the presence of EDP, suggesting an uncoupling of EDP signaling in aged fibroblasts. Several molecular alterations in ERC-induced signaling pathways has been previously describe such as modification of Gi protein activation [33], alteration of calcium signaling [60,61] and modulation of ERK1/2 phosphorylation [62]. From these data, we hypothesized that the uncoupling of ERC involved a modification of upstream molecular events.

It has been shown that alteration of EDP signaling with age does not imply a modification of EBP expression and affinity [34]. Moreover, we show that aging does affect neither the mRNA expression of the ERC subunits nor the protein levels of PPCA and Neu-1. We therefore concluded that mRNA or protein expression of the receptor sub-units could not account for the age-related uncoupling of ERC.

Accordingly, we supposed that age-related alterations in EDP signaling would involve the first steps of their signaling cascade. Indeed, modifications of Neu-1 sialidase activity or plasma membrane composition could explain the age-related uncoupling of ERC. Gangliosides are sialic acid-containing glycosphingolipids found in microdomains of the plasma membrane. They are involved in cellular interactions and in signal transduction processes [51,52]. Binding of EDP to EBP triggers Neu-1 sialidase activation leading to the conversion of GM_3_ ganglioside to LacCer, which behaves as a second messenger and transduces EDP signaling [28,29]. Our results evidenced an increase in basal sialidase activity with age but also an alteration of EDP-induced sialidase activity. Such variation has been already reported by Saito *et al* who described age-dependent modulations of sialidase activity in synaptic plasma membrane isolated from mice brain [63]. Moreover, we observed an increase of basal LacCer production levels as a function of age. Strikingly, we observed that this age-related LacCer production could be due to increased sialidase activity as the use of DANA significantly reduced LacCer production. Interestingly, LacCer production following EDP treatment was altered during aging. The ability of EDP to promote LacCer formation was lost in aged fibroblasts as compared to young and intermediate ones. The use of DANA suggested that Neu-1 was involved in this EDP-mediated LacCer production.

Overall, our data suggest, for the first time, that a modification of basal Neu-1 activity and the subsequent alteration in LacCer production could be responsible for the alteration of EDP signaling observed with aging. Moreover, as Neu-1 is involved in elastogenesis through the desialylation of microfibrils [39,64], an EBP-tropoelastin bound dependent process, our data point out that the deregulation of its activity during aging could partly explain the difficulty encountered to achieve an efficient elastogenesis in aged individuals.
